# Effect of Ultraviolet Aging on Rheology and Chemistry of LDH-Modified Bitumen

**DOI:** 10.3390/ma8085238

**Published:** 2015-08-12

**Authors:** Xing Liu, Shaopeng Wu, Gang Liu, Liping Li

**Affiliations:** State Key laboratory of Silicate Materials of Architectures, Wuhan University of Technology, Wuhan 430070, China; E-Mails: liuxing1107@whut.edu.cn (X.L.); lipingli@whut.edu.cn (L.L.)

**Keywords:** LDHs, bitumen, aging, rheology, chemical compositions

## Abstract

Layered double hydroxides (LDHs) are an ultravioletlight (UV)-resistant material. In this study, two types of LDHs (Mg-Al-LDHs and Zn-Al-LDHs) were applied to modify bitumen by melt-blending. The effect of ultraviolet aging on the rheology and chemistry of LDH-modified bitumen was studied by means of dynamic shear rheometer (DSR), thin-layer chromatography with flame ionization detection (TLC-FID), Fourier transform infrared spectroscopy (FTIR), and Ultraviolet-Visible (UV-Vis) spectrophotometry to reveal the mechanisms of action for LDHs and bitumen. The results showed that within the UV spectra (220–400 nm), the reflectance of Zn-Al-LDHs was larger than that of Mg-Al-LDHs. These two LDHs have different influences on the performance of bitumen. Mg-Al-LDHs had a more obvious influence on the physical and dynamic rheological properties of bitumen than Zn-Al-LDHs. Zn-Al-LDHs improved the UV-aging resistance of bitumen more. The reason can be that the reflectance of the Zn-Al-LDHs to the UV light is larger than that of the Mg-Al-LDHs. The Zn-Al-LDH-modified bitumen had more potential to improve the UV-aging resistance during the service life of asphalt pavement.

## 1. Introduction

Bitumen aging is one of the main reasons for asphalt pavement failure. Normally, aging effects on bitumen are classified into two types: thermal-oxidative aging and light-oxidative aging. The thermal-oxidative aging refers to aging during mixing, storage, transportation, laying, and compaction of the mixture; the light-oxidative aging (UV aging) takes place during the service life of the asphalt mixture. Both of them can change the physical and chemical characteristics, causing the pavement to be stiff and brittle and consequently shorten its service life [1,2,3]. To prevent bitumen from UV aging, one effective way is bitumen modification. Many modifiers have been investigated to improve the aging resistance of the bitumen, like organo-montmorillonite (OMMT), carbon black, *etc.* [4,5,6,7].

Layered double hydroxides (LDHs) have attracted considerable attention as an anti-UV agent in recent years. They belong to the class of anionic layered materials formed by interlayer anions and laminates with a positive charge. LDHs have a multi-nestification layered structure. The inorganic layer sheets can function as a shield to physically prevent the UV light; metal atoms of layer sheets and negative ions between layer sheets can chemically absorb the UV light [8,9,10,11,12]. Due to its fundamental structure, LDHs have the potential to improve the UV-aging resistance of bitumen. In recent years, LDHs have been used as additives to alleviate the UV-aging effect of bitumen. Results showed that LDHs can enhance the UV-aging resistance of bitumen due to its high UV reflectance of LDHs with a multilayered structure [13,14,15]. However, there is no research to compare the influence of different LDHs on the UV-aging resistance of bitumen.

In this study, two types of LDHs were used to modify the bitumen by melt-blending. The effect of ultraviolet aging on the rheology and chemistry of LDH-modified bitumen was studied. In order to evaluate the UV-aging resistance of LDH-modified bitumens, a Ultraviolet-Visible (UV-Vis) spectrophotometer was adopted to characterize the reflectance of LDHs. The dynamic shear rheometer (DSR) test was used to investigate the rheological characteristics of the binders before and after UV aging. Then, the Fourier transform infrared (FTIR) spectroscopy and thin-layer chromatography with flame ionization detection (TLC-FID) methods were used to compare the functional groups and chemical compositions of the binders, respectively. Finally, the physical properties were also investigated to evaluate the UV-aging resistance of all the binders.

## 2. Materials and Test Methods

### 2.1. Materials and Preparation

**Figure 1:**
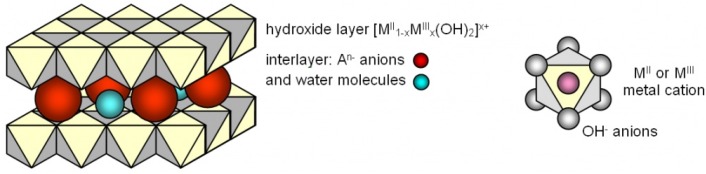
The structure of LDHs.

Base bitumen was provided by SK Corporation, Ulsan, Korea, with the penetration value of 87 dmm at 25 °C, the softening point of 49.4 °C, and the dynamic viscosity of 330 Pa·s at 60 °C. Two types of LDHs were provided by RuiFa Chemical Company Ltd., Jiangyin, China. Mg-Al-LDHs and Zn-Al-LDHs were a white powder. The molecular formula of the LDHs was as follows:

M^II^_1−x_M^III^_x_(OH)_2_(CO_3_)_x/2_·mH_2_O

where, x is the content variable of metallic elements, with 0.2 ≤ x ≤ 0.33; m is the amount of crystal water, with 0 ≤ m ≤ 2. Figure 1 shows the structure of LDHs.

To prepare the LDH-modified bitumen sample, the base bitumen was first heated to be fluid at around 140 °C; then, LDH powders (3 wt % Mg-Al-LDHs and 3 wt % Zn-Al-LDHs in this study) were slowly added to the base bitumen and sheared for 1 h by using a high-speed shearing mixer at a shearing speed of 4000 rpm and 140 °C. The base bitumen was also exposed to the same shearing process to be a reference.

### 2.2. Procedure of UV Aging

First, the thin film oven test (TFOT) was performed according to American Society for Testing and Materials (ASTM) D 1754 to simulate the short-term aging of bitumen during the mixing process of the asphalt mixture [16]. Next, the UV photo-oxidation test was measured in a UV-aging oven with the testing temperature of 50 °C and the UV strength of 8000 μW·cm^−2^ for 4 days. The thickness of the film sample was 1250 μm. The detailed procedure was as follows: to prepare the bitumen film with a thickness of 1250 μm, 19 grams of bitumen, after the short-term aging, were poured into a steel plate with a diameter of 140 mm. After the UV aging, the bitumen sample in the plate was heated in the oven at 140 °C and mixed by a glass bar. Then, the sample was poured out on the silicate paper. After cooling down, it was used for the test.

### 2.3. Rotational Viscometer and DSR Test

Rotational viscometer (DV- ǁ + Pro, Brookfield, Middleboro, MA, USA) was used to test the viscosity of the samples according to the standard ASTM D4402-06 [17]. The testing temperature was set at 135 °C.

Dynamic Shear Rheometer (DSR) (MCR 101, Anton Paar Company, Graz, Austria) was used to measure the dynamic rheological properties of the base and modified bitumens before and after UV aging. The temperature sweep test was from −10 to 40 °C with the increment of 2 °C per minute, which was performed under the strain-controlled mode at a constant frequency of 10 rad·s^−1^. Moreover, the diameter of the plate was 8 mm, and the gap between the plates was 2 mm.

### 2.4. UV-Vis Test

Ultraviolet-Visible (UV-Vis) spectroscopy Lambda 750S (PerkinElmer Company, Waltham, MA, USA) was adopted to characterize the reflectance of LDHs. Polytetrafluoroethylene (Aifusi Chemical Company Ltd., Yangzhong, China) was used as a standard in the UV-Vis experiment. The wavelength range was selected within the region of 200–800 nm.

### 2.5. TLC-FID Test

Iatroscan MK-6 analyzer (Iatron Laboratories Inc., Tokyo, Japan) was used to measure the chemical compositions of the base and modified bitumens before and after UV aging. Sample solutions with concentration of 2% (w/v) were prepared by dissolving 80 mg bitumen in 4 mL dichloromethane. After chromarods were cleaned and activated in FID-flame, 1 μL of the solution was spotted on the chromarod using a spotter. The separation of bitumen fractions was performed with a three-stage process. The first development was in n-heptane (70 mL) and expanded to 100 mm of the chromarods, the second stage in toluene/n-heptane (70 mL, 4/1 by volume) was developed to 50 mm, and the last development was in toluene/ethanol (70 mL, 11/9 by volume) and expanded to 25 mm. The solvent was evaporated in an oven at 80 °C after each stage. Then the chromarods were scanned in the TLC-FID analyzer. Five chromarods were tested for each sample, and finally the average value of five readings was used as the result.

### 2.6. FTIR Test

Fourier Transform Infrared (FTIR) spectroscopy (Nexus, ThermoNicolet Corp., Waltham, MA, USA) is a technique used to identify functional groups in organic compounds at a molecular level. We dissolved a 5% bitumen sample by weight in carbon disulfide. Then, a certain amount of solution was dropped onto a KBr stage and dried by the infrared lamp for a moment to form a thin film specimen.

### 2.7. Physical Properties Test

To evaluate the physical properties of bitumen samples before and after aging, penetration (25 °C) and softening point (ring and ball method) were investigated according to the standard ASTM D5 [18] and ASTM D36 [19], respectively.

## 3. Results and Discussions

### 3.1. Reflectance of LDHs

**Figure 2:**
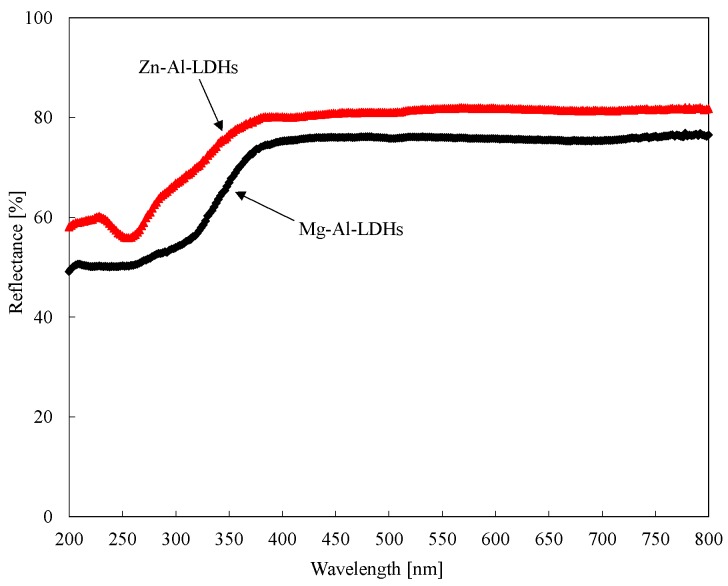
Reflectance of two types of LDHs.

Figure 2 shows the reflectance of the two LDHs used in this study. Obviously, both LDHs had a high reflectance to the light with a wavelength ranging from 200 to 800 nm. Within the UV spectra region (220–400 nm), the reflectance of Zn-Al-LDHs was larger than that of Mg-Al-LDHs, indicating that Zn-Al-LDHs had more ability to prevent UV light.

The zinc atoms of Zn-Al-LDHs can combine with an oxygen atom placed in between the layers to form zinc oxide (ZnO), which has a wider band gap (3.37 eV) than magnesium oxide (MgO). Thus, this structure can better absorb UV light and convert it to heat and light when compared to Mg-Al-LDHs [20,21,22]. Therefore, Zn-Al-LDHs has more ability to shield the UV light.

### 3.2. Effect of UV Aging on Rheology of LDH-Modified Bitumen

#### 3.2.1. Dynamic Viscosity

The dynamic viscosities of base and modified bitumens before and after UV aging are given in Table 1. As indicated, the addition of two types of LDHs increased the dynamic viscosity of the base bitumen. After the UV aging, the dynamic viscosity of each bitumen also increased. The base bitumen showed the largest value of dynamic viscosity after UV aging, and the Zn-Al-LDH-modified bitumen showed the smallest value of viscosity. The viscosity aging index (VAI) was calculated by Equation (1) and used to evaluate the aging degree of bitumen:

VAI = (V_2_ − V_1_)/V_1_ (1)

where, V_1_ and V_2_ are dynamic viscosities of bitumen before and after UV aging at 135 °C, respectively. Obviously, the VAI value for LDH-modified bitumen was lower than that for the base bitumen. The VAI value of Zn-Al-LDH-modified bitumen was lower than that of Mg-Al-LDH-modified bitumen, and exhibited the better performance of UV-aging resistance.

**Table 1:** Dynamic viscosity of base and modified bitumens before and after UV aging at 135 °C.

Type of Bitumen	V_1_ of Fresh Bitumen (Pa·s)	V_2_ of Bitumen after UV Aging (Pa·s)	VAI *
Base	0.325 ± 0.001	2.786 ± 0.002	7.6
Mg-Al-LDHs + Base	0.451 ± 0.002	1.395 ± 0.002	2.1
Zn-Al-LDHs + Base	0.430 ± 0.002	1.262 ± 0.002	1.9

* VAI was calculated by using the average of V_1_ and V_2_ which were tested with three repetitions.

#### 3.2.2. Complex Modulus and Phase Angle

**Figure 3:**
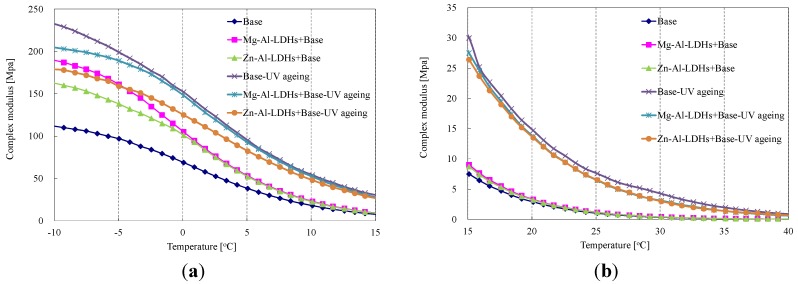
Effect of different LDHs on G* of SK-90 bitumen before and after UV aging.

Figure 3 shows the effect of the two LDHs on the complex modulus (G*) of SK-90 bitumen before and after UV aging. The higher the value of G*, the stiffer the bitumen [23]. As indicated in Figure 3, both LDHs can increase the values of G*, especially at a lower temperature below 0 °C. Mg-Al-LDHs had more ability to increase G* than Zn-Al-LDHs, but both LDHs had the same influence on G* when the temperature was higher than 0 °C. Moreover, after UV aging, the G* values of modified bitumens were smaller than that of the base bitumen. The G* value of Zn-Al-LDH-modified bitumen exhibited a lower value than that of Mg-Al-LDH-modified bitumen within the temperature range from −10 to 0 °C, indicating that Zn-Al-LDH-modified bitumen exhibited a better aging resistance.

**Figure 4:**
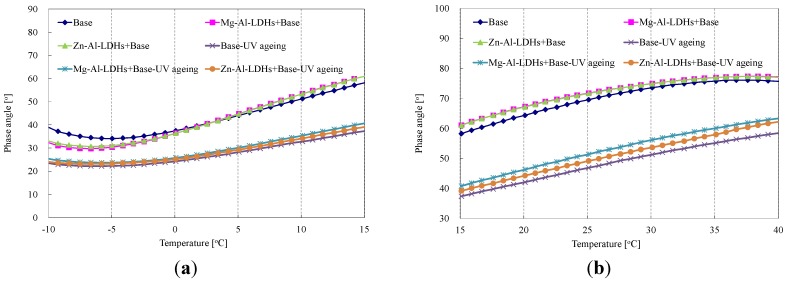
Effect of different LDHs on phase angle of SK-90 bitumen before and after UV aging.

Figure 4 shows the effect of different LDHs on the phase angle of SK-90 bitumen before and after UV aging. Phase angle (*δ*) represents the viscoelastic character of bitumen, and a smaller *δ* value means more elasticity [24]. With the addition of two types of LDHs, the *δ* values became smaller than the base bitumen at the low temperature, then larger than the base bitumen when the temperature exceeded 2.5 °C (see Figure 4), indicating a property transformation of bitumen from viscosity to elasticity at the low temperature (−10–2.5 °C) and from elasticity to viscosity at the medium temperature (2.5–40 °C). After UV aging, the *δ* values of base and modified bitumens decreased with the temperature range from −10 to 40 °C, indicating that the UV aging decreased the phase angle value. As shown in Figure 4, the *δ* values of modified bitumens were larger than that of base bitumen after UV aging. The Zn-Al-LDH-modified bitumen had a smaller decrease within the temperature range from −10 to 40 °C, resulting in the largest *δ* value. Therefore, the largest and smallest change level of viscoelastic transformation from viscosity to elasticity were the base bitumen and Zn-Al-LDH-modified bitumen, respectively. This corresponded well with the viscosity result that the LDH-modified bitumens improved the UV-aging resistance of bitumen, especially the Zn-Al-LDH-modified bitumen.

### 3.3. Effect of UV Aging on Chemistry of LDH-Modified Bitumen

#### 3.3.1. Analysis of TLC-FID

Table 2 gives the chemical compositions of base and modified bitumens before and after UV aging. With the addition of different LDHs, the contents of four chemical compositions showed slight changes incidentally. The colloidal index (Ic) was calculated by Equation (2):

Ic = (aromatics + resins)/(saturates + asphaltenes)
(2)

A higher value of Ic means that the asphaltenes are more peptized by the resins in the oil-based medium, indicating a more homogeneous sol system [25]. As shown in Table 2, LDH-modified bitumen had a higher Ic value, especially with Zn-Al-LDHs. The four-chemical compositions and Ic values of modified bitumen were changed after UV aging. All binders exhibited a decrease in aromatics, an increase in asphaltenes, and a slight change in saturates and resins, which definitely caused a reduction of the Ic value. Compared to all binders, the base bitumen showed the largest decrease, while the Zn-Al-LDH-modified bitumen showed the smallest the decrease. The reduction of the Ic value indicates a chemical transformation of bitumen from sol to gel as a result of UV aging [26]. The Mg-Al-LDHs and Zn-Al-LDHs affected the transformation to a different extent. Obviously, both of them reduced the rate at which the bitumen transforms from sol to gel, indicating that the two types of LDHs restrained the gelation process of bitumen during the UV aging, especially Zn-Al-LDHs. This corresponded well with the viscosity and complex modulus result that Zn-Al-LDH-modified bitumen had better UV-aging resistance of bitumen.

**Table 2:** Chemical compositions of base and modified bitumens before and after UV aging.

Type of Bitumen	Saturates (%)	Aromatics (%)	Resins (%)	Asphaltenes (%)	Ic
Base-fresh	11.88	36.78	43.02	8.32	3.95
Mg-Al-LDHs + Base-fresh	12.53	37.20	42.99	7.28	4.05
Zn-Al-LDHs + Base-fresh	12.20	35.82	44.80	7.18	4.16
Base-UV aging	12.13	25.74	41.41	20.72	2.04
Mg-Al-LDHs + Base-UV aging	11.39	30.10	40.34	19.77	2.26
Zn-Al-LDHs + Base-UV aging	10.96	26.73	43.77	18.54	2.39

#### 3.3.2. Analysis of FTIR

Infrared (IR) spectra can indicate the existence or absence of chemical functional groups. During the aging process of bitumen, the changes of carbonyl (at 1700 cm^−1^) and sulphoxide (at 1030 cm^−1^) are frequently used. In this study, the area of the spectra bands between 600 cm^−1^ and 2000 cm^−1^ was chosen as the reference [27]. For quantitative analysis, two structural indexes are calculated based on the band area according to Equations (3) and (4):

I_C=O_ = A_1700 cm_^−1^/ΣA (3)

I_S=O_ = A_1030 cm_^−1^/ΣA (4)

where, A_1700 cm_^−1^ is the area of the carbonyl band centered on 1700 cm^−1^ (calculated from 1650–1750 cm^−1^), A_1030 cm_^−1^ is the area of the sulphoxide band centered on 1030 cm^−1^ (calculated from 980–1080 cm^−1^), and ΣA is the area of the spectra bands between 600 cm^−1^ and 2000 cm^−1^.

**Figure 5:**
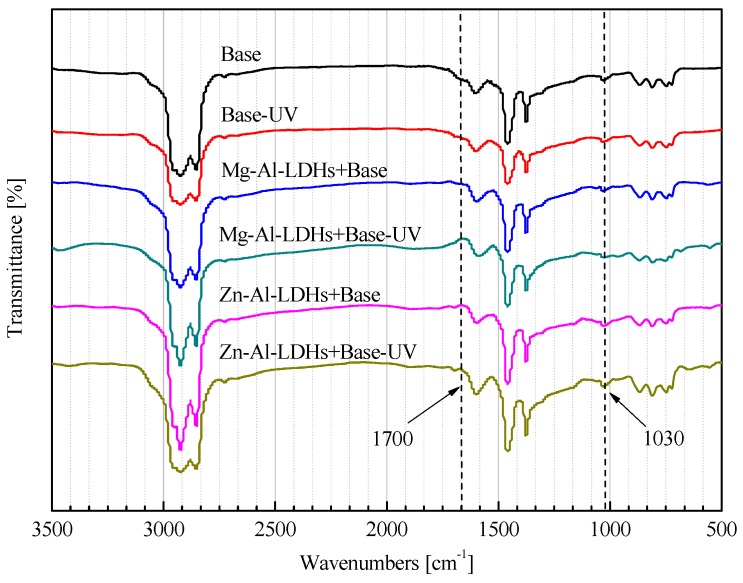
FTIR spectra of base and LDH-modified bitumens before and after UV aging.

Figure 5 shows the FTIR spectra of base and LDH-modified bitumens before and after UV aging. The UV-aging process can accelerate the oxidation of bitumens, and increase the peak area of carbonyl at 1700 cm^−1^ and sulphoxide at 1030 cm^−1^. The structure-change indexes of base and modified bitumens are given in Table 3. After UV aging, the carbonyl index (I_C=O_) of the base bitumen increased by 0.0164, and the sulphoxide index (I_S=O_) by 0.0340. However, the I_C=O_ values of Mg-Al-LDH- and Zn-Al-LDH-modified bitumens only increased by 0.0143 and 0.0103, and the I_S=O_ by 0.0265 and 0.0160, respectively. Therefore, the addition of two types of LDHs can inhibit the oxidation of bitumen during UV aging. The inhibition is the most important in the Zn-Al-LDH-modified bitumen.

**Table 3:** Structure-change indexes of base and modified bitumens before and after UV aging.

Sample	Index	Fresh	UV Aging
Base	I_C=O_	0.0036	0.0200
	I_S=O_	0.0100	0.0440
Mg-Al-LDHs + Base	I_C=O_	0.0036	0.0179
	I_S=O_	0.0100	0.0356
Zn-Al-LDHs + Base	I_C=O_	0.0037	0.0140
	I_S=O_	0.0100	0.0260

### 3.4. Effect of UV Aging on Physical Properties of LDH-Modified Bitumen

Physical properties such as penetration and softening point are basic and direct indexes to evaluate the properties of bitumen. The penetration retention rate (PRR) that is calculated by Equation (5) is often used to evaluate the aging degree of bitumen:

PRR = P_2_/P_1_ × 100% (5)

where P_1_ and P_2_ are the penetration of bitumen before and after UV aging, respectively. The softening point increment (SPI) that is calculated by Equation (6) is often used to evaluate the aging degree of bitumen:

SPI = SP_2_ − SP_1_ (6)

where SP_1_ and SP_2_ are the softening points of bitumen before and after UV aging, respectively. Physical properties of base and modified bitumens before and after UV aging are shown in Table 4. With the addition of Mg-Al-LDHs and Zn-Al-LDHs, the softening point of the bitumen was increased while the penetration was decreased because the LDHs made the bitumen more viscous, and the Mg-Al-LDHs had a more obvious influence on the physical properties. After UV aging, the softening point of all binders increased significantly but penetration decreased. Among all the binders, the Zn-Al-LDH-modified bitumen showed the largest value in PRR and the smallest value in SPI, indicating that Zn-Al-LDHs had a minor aging effect. Therefore, it can be concluded from Table 4 that LDH-modified bitumen enhances the UV-aging resistance of pavement during its service life, especially Zn-Al-LDH-modified bitumen.

**Table 4:** Physical properties of base and modified bitumens before and after UV aging.

Type of Bitumen	Penetration (25 °C, dmm)		PRR * (%)	Softening Point (°C)		SPI * (°C)
	Fresh	UV Aging		Fresh	UV Aging	
Base	87 ± 1	53 ± 2	61	49.4 ± 0.2	66.9 ± 0.2	17.5
Mg-Al-LDHs + Base	68 ± 2	56 ± 1	82	51.8 ± 0.2	62.5 ± 0.4	10.7
Zn-Al-LDHs + Base	72 ± 1	61 ± 2	85	51.4 ± 0.2	59.5 ± 0.2	8.1

* PRR and SPI were calculated by using the average of penetration and softening point before and after UV aging which were tested with three repetitions, respectively.

## 4. Conclusions

LDH-modified bitumen was prepared by the melt-blending method. According to the test results, LDHs had a significant influence on the rheology and chemistry of the base bitumen before and after UV aging. Some conclusions can be drawn as follows:

The viscosity and complex modulus of LDH-modified bitumens were increased, but the phase angle was decreased after the UV aging. Mg-Al-LDHs had a more obvious influence on the physical and dynamic rheological properties of bitumen than Zn-Al-LDHs. However, Zn-Al-LDHs improved the UV-aging resistance of bitumen more significantly. The reason can be that the reflectance of Zn-Al-LDHs to UV light is larger than that of Mg-Al-LDHs within the UV spectra (220–400 nm). In comparison, the Zn-Al-LDH-modified bitumen had more potential to improve the UV-aging resistance during the service life of the asphalt pavement.
